# ALIAmides Update: Palmitoylethanolamide and Its Formulations on Management of Peripheral Neuropathic Pain

**DOI:** 10.3390/ijms21155330

**Published:** 2020-07-27

**Authors:** Ramona D’Amico, Daniela Impellizzeri, Salvatore Cuzzocrea, Rosanna Di Paola

**Affiliations:** 1 Department of Chemical, Biological, Pharmaceutical and Environmental Sciences, University of Messina, Viale Ferdinando Stagno D’Alcontres 31, 98166 Messina, Italy; rdamico@unime.it (R.D.); dimpellizzeri@unime.it (D.I.); dipaolar@unime.it (R.D.P.); 2 Department of Pharmacological and Physiological Science, Saint Louis University School of Medicine, 1402 South Grand Blvd, St Louis, MO 63104, USA

**Keywords:** peripheral neuropathic pain, neuroinflammation, ALIAmides, palmitoylethanolamide

## Abstract

Neuropathic pain results from lesions or diseases of the somatosensory nervous system and it remains largely difficult to treat. Peripheral neuropathic pain originates from injury to the peripheral nervous system (PNS) and manifests as a series of symptoms and complications, including allodynia and hyperalgesia. The aim of this review is to discuss a novel approach on neuropathic pain management, which is based on the knowledge of processes that underlie the development of peripheral neuropathic pain; in particular highlights the role of glia and mast cells in pain and neuroinflammation. ALIAmides (autacoid local injury antagonist amides) represent a group of endogenous bioactive lipids, including palmitoylethanolamide (PEA), which play a central role in numerous biological processes, including pain, inflammation, and lipid metabolism. These compounds are emerging thanks to their anti-inflammatory and anti-hyperalgesic effects, due to the down-regulation of activation of mast cells. Collectively, preclinical and clinical studies support the idea that ALIAmides merit further consideration as therapeutic approach for controlling inflammatory responses, pain, and related peripheral neuropathic pain.

## 1. Introduction

The International Association for the Study of Pain (IASP) describes pain as “an unpleasant sensory and emotional experience that is associated with real or probable tissue damage, as defined in rapports of such injury” [1]. Although it is easy to conceptualize pain as a homogeneous entity, in reality there are several different types, each with distinct neurobiological and pathophysiological mechanisms.

Pain can be categorized in numerous ways: acute or chronic based on duration; cutaneous, deep or superficial, muscle, or visceral based on location; and, inflammatory, neuropathic, or cancer based on cause or type. The main classification divides pain into neuropathic and nociceptive [2]. This distinction is necessary, because it reflects not only the origin of pain, but gives information about the treatment. As for nociceptive, it is typically well localized due to the high concentration of nociceptors in somatic tissues [3]. Instead, the IASP describes neuropathic pain as “pain caused by somatosensory nervous system damage or disease” [4]. This definition is broad and encompasses more than 100 conditions, and it includes injuries that span the entire neuro-axis of pain [5]. In fact, neuropathic pain is not a particular disease, but a condition that is caused by a variety of different diseases and lesions that manifest as a series of symptoms and complications.

Neuropathic pain, in turn, is divided into two classes: central and peripheral neuropathic pain, depending on the site of the lesion that is causing the pain. Table 1 summarizes the more common neuropathic pain. Central neuropathic pain is triggered by spinal cord and/or brain damages or diseases, such as central post-stroke pain (CPSP) and neurodegenerative disorders, particularly Parkinson’s disease, and it affects the central somatosensory pathway [6].

Conditions of central neuropathic pain will likely have several underlying mechanisms and warrant separate consideration. This review focuses on conditions affecting peripheral neuropathic pain, which originate from injury to the peripheral nervous system (PNS). There are multiple avenues for peripheral nerve damage, including mechanical, chemical, and infectious.

The mechanisms underlying these different conditions are multiple. Some of the mechanisms are well known, but many are not. For this reason, a greater understanding of the mechanisms of pain, how it is activated, and how information is transmitted to the CNS should put us in a better position to treat patients and design rational treatment strategies.

Pain is transmitted to the spinal cord by various receptors, like thermoreceptors, mechanoreceptors, chemoreceptors, pruriceptors, and nociceptors. These are specialized primary afferent neurons that are capable of detecting noxious peripheral stimuli [7] from joints, muscles, skin, and send these signals to the spinal cord and eventually to the brain for more processing [8]. Looking from a cellular/molecular aspect, transmission of nociceptive signals within the CNS is regulated by several cellular and intracellular elements [9,10], as described in Table 2:

When a noxious stimulus activates an ion channel on a nociceptor, such as the activation of acid-sensing ion channels (ASIC) by protons, cation influx depolarizes the nociceptor producing a potential receptor. If the receptor potential is of sufficient magnitude to reach the voltage-gated Na channel (NaV) activation threshold, then this will trigger potential action generation and pain transmission to the spinal cord [11,12].

Once damage occurs, inflammation and reparatory processes result in a hyperexcitable condition, called peripheral sensitization, leading to a decrease in the threshold of stimulus (pain) for nociceptor activation [13]. Many conditions can contribute to peripheral sensitization. Among these, proinflammatory cytokines are also widely involved in sensitization of the PNS and may contribute to allodynia and hyperalgesia (see Table 3) [14,15], which are common features in all peripheral pains [16,17].

## 2. Neuroinflammation

Recent research has shown that neuroinflammation plays a key role in the peripheral neuropathic pain [18,19,20,21]. In both CNS and PNS, the inflammatory events occur at different levels than those in other tissues and they involve multiple types of cells [22,23]; such observations contribute to the introduction of the name “neuroinflammation”. The latter is controlled by a complex network of regulatory mechanisms that limit the potentially dangerous effects of persistent inflammation. Nonetheless, when neuroinflammation is prolonged, it overcomes the limits of physiological control and induces harmful effects, including pro-inflammatory signalling pathways, increased oxidative stress, and the death of neurons linked to chronic pathogenesis of neuropathic pain [24,25]. For these reasons, neuroinflammation in both the CNS and PNS plays a central role in the pathogenesis of neuropathic pain [26], as well as chronic neurodegenerative diseases [27,28,29], psychiatric illness [30], and even autism spectrum disorder [31].

Several excellent reviews concentrate on the role of microglia in neuropathic pain [20,32,33,34,35]. Microglia play an active part in preserving normal physiological conditions, as it perceives the cellular surroundings with its ramified processes and undergoes quick morphological changes in response to different stimuli [36,37,38], including peripheral nerve injury (PNI). Microglial activation consists in changes in morphology (from ramified to amoeboid), migration to the site of injury, increases in the expression of microglial markers, such as CD11b and Iba1, and, in addition, increased its proliferation [39,40]. Glial cell proliferation is rarely observed under normal circumstances; on the contrary, a substantial microglial proliferation occurs under some neuropathic pain circumstances, such as after compression of the sciatic nerve, partial sciatic nerve ligation, or spared nerve injury [41]. In addition to the morphological changes that follow nerve injury, there are also substantial biochemical alterations, important for microglia in pain induction. Microglial activation that is caused by nerve damage results in substantial up-regulation of the expression of the ATP receptor P2X4 and of the chemokine receptor CX3CR1 in spinal cord microglia [42,43,44]. The spinal blockage of the signals P2X4 and CX3CR1 has been shown to attenuate neuropathic pain that is caused by nerve damage [42,45,46]. In addition, the phosphorylation of p38 mitogen-activated protein kinase (MAPK) in spinal cord microglia has also been shown to cause nerve damage [47,48]. This implies a significant increase in the levels of phsopho-p38 (p-p38), which, in physiological conditions, it is found in low concentrations [49,50]. These are just a few of the possible microglial biochemical changes induced by nerve-injury that may be involved in generating and maintaining neuropathic pain. Despite the uncertain mechanism by which microglial cells cause neuropathic pain, multiple studies have shown that microglial activation inhibiting decreases hyperalgesia and allodynia following nerve damage [51,52,53].

Although glial cell activation is widely accepted as contributing to neuropathology, it should not be forgotten that microglia (and also astrocytes) also respond to pro-inflammatory signals that are released from other immune cells. Comprehension of the role of the immune system in neuroinflammation became clear with the recognition that an extensive communication exists between the immune system itself and CNS. In this perspective, mast cells provide a potentially important peripheral immune signalling connection to the brain in the inflammatory setting [54]. Mast cells are first responders to intervene as recruiters to initiate, amplify, and prolong all of the immune and nerve responses that arise from their activation [55]. Studies demonstrate that the degranulation of mast cells can produce factors that sensitise nociceptors, thus directly contributing to neuropathic pain [54,56,57]. Furthermore, mast cells can move through the blood–brain barrier (BBB), but also through the blood-spinal cord barrier (BSCB), both in normal circumstances and disease states [58]. This increase in the permeability of the BBB and blood-spinal cord barrier (BSCB) leads to increased leukocyte invasion to the CNS and PNS [22]. Vascular changes, together with leucocytes infiltration, are the basis of the pathophysiology of peripheral neuropathic pain. Therefore, multiple alterations of vascular, metabolic, and autoimmune origin involve oxidative and nitrosative stress, neuroinflammation, microvascular ischemia, altered peripheral nervous tissue, angiogenesis, and neuroanatomical changes, which provoke the formation of endoneurial edema and the release of reactive oxygen species (ROS) [59,60].

The observations that mast cells and microglia are frequently involved at similar sites after nerve injury or inflammation has led to speculation as to whether both cell types may represent the chief actors in the regulation of inflammatory pain. In addition to microglia and MC, other cells play an important role in mediating neuroinflammation astrocytes, oligodentrocytes, inflammasomes, cytokines, and chemokines (see Table 4).

The fact that multiple factors can sensitize nociceptors may partly explain why it is difficult to exactly quantify how common neuropathic pain is due to problems with the definition and assessment of neuropathic pain, but about a fifth of people reporting chronic pain have primarily neuropathic pain. Epidemiological studies report that prevalence in the general population is estimated between 7% and 10% [13], while, in Europe, the incidence of chronic pain is around 25–30% [69,70].

One motive for the high prevalence rate of chronic pain, and particularly neuropathic pain, is the lack of efficient treatments. The primary reason for that is the incapacity to target precisely mechanisms that generate pain. In fact, syndromes that lack distinct pathophysiological mechanisms, such as fibromyalgia, incline to be associated with high rates of treatment failure in pain [71]. In additional, it is good to specify that pain is a multidimensional experience, which involves psychological and sociocultural factors, such as depression, somatization, social stress, and negative job satisfaction. All of these factors can contribute to the onset of chronic pain after an acute episode [72,73,74].

This has led to considering neuropathic pain not only a medical problem, but also a socio-economic distress that requires urgent attention.

## 3. Pharmacotherapy in Peripheral Neuropathic Pain

Given the risks that neuroinflammation poses to the body, it is not surprising that much effort is devoted to developing efficacious pharmacological interventions. This review will give a brief overview of the therapeutic strategies that are currently in use in neuropathic pain and then discuss novel approaches for counteracting neuroinflammation, which are based on endogenous defence mechanisms and lipid signaling molecules. First-line drugs for neuropathic pain include antidepressants and anticonvulsants acting at calcium channels [75]. Second- and Third-Line Drugs for neuropathic pain include topical analgesics, opioids, and corticosteroids [75]. Other treatment modalities in the management of neuropathic pain include non-pharmacologic therapies and interventions, which can be considered as adjunctive agents to pharmacotherapy in appropriate patients. Table 5 summarizes the pharmacotherapies in use in neuropathic pain.

## 4. Overview on ALIAmides

One of the most widely studied families of molecules in recent years in the context of neuroinflammation is the family of ALIAmides, autacoid local injury antagonist amides. The term autacoids refers to endogenous compounds or the precursors or other derivatives thereof, they are produced on request, and then metabolized in the same cells and/or tissues [94]. Because autacoids are endogenous molecules, they provide a number of benefits over the treatment with traditional drugs. Primarily, metabolic pathways are intrinsic to the tissue and this means no production of toxic metabolites. Moreover, classical drugs focus on blocking one target receptor only, which lead to a sudden halt of a physiological process and lead to collateral damage. On the contrary, instead modern autacoid medicine looks at endogenous compounds or their derivatives, which use physiological pathways to modify pathological processes, so the probability of side effects is low [95,96]. In 1993, the Nobel laureate Rita Levi-Montalcini coined the term “aliamides” for such compounds [97]; they represent a small host of naturally occurring N-acyl ethanolamines (NAEs) that are particularly enriched in animal tissues [98]. Levi-Montalcini was the first scientist to discover that the tissue accumulation of NAE occurred under pathological degenerative conditions, and this is an important biological response to control such inflammation. In fact, the main mechanism of action of ALIAmides mainly relies on the down-modulation of cell hyperactivity following injury. Their main cellular targets are mast cells (MCs), whose behaviour, proliferation, and function are indeed under aliamide control [99,100,101]. In mammals, aliamides are produced “on demand” by tissues, i.e., enzymatically released from membrane precursors when cells face potentially noxious stimuli, and they are principally metabolized by intracellular hydrolases [102]. ALIAmides represent a group of endogenous bioactive lipids, including palmitoyl ethanolamide (PEA), oleoyl ethanolamide (OEA), and stearoyl ethanolamide (SEA), which play a central role in numerous biological processes, including pain, inflammation, and lipid metabolism [103,104]. Thus, ALIAmides or their analogues are emerging as possible therapeutic strategies in the treatment of numerous chronic inflammatory conditions, such as pain [105,106] and tissue inflammation [101,107].

Below, we will make a brief overview of the ALIAmides and then deepen their role, in particular of PEA, in peripheral neuropathic pain.

### 4.1. PEA

Many compounds have been described, which act as ALIAmides; however, PEA is considered to be the first ALIAmide and the most studied. Its anti-inflammatory and immune-modulating properties were described in 1957 after isolation from egg yolk [108,109]. However, it was the crucial work of the Nobel laureate Rita Levi-Montalcini that rekindled attention in this molecule in the 1990s [110], helping to reveal its powerful anti-inflammatory and anti-nociceptive effects. The potential therapeutic use of PEA has led many researchers to identify other natural sources that are rich in this compound. In fact, PEA has also been found in the seeds of some varieties of legumes, such as peas and beans, as well as in some varieties of vegetables, such as tomatoes and potatoes. Finally, high levels of PEA were also found in human, bovine, and moose milk [111,112,113].

In some European countries, PEA is actually marketed for veterinary use (skin conditions, RedonylTM, (Innovet)) and as a nutraceutical in humans (NormastTM, PelvilenTM [Epitech]). PEA has shown high safety and tolerability [114,115], and it is particularly used in humans because its anti-inflammatory and analgesic properties [116,117]. PEA has been suggested to act as a protective endogenous mediator that is produced “on demand” during inflammatory and neurodegenerative conditions to counter inflammation, pain, and neuronal damage. In fact, it down-modulates the activation of mast cells and microglia [118]. In addition, multiple studies have demonstrated that the PEA rates are increased in brain regions involving nociception and spinal cord following neuropathic pain induction and other stroke-related conditions [55].

**Figure 1:**
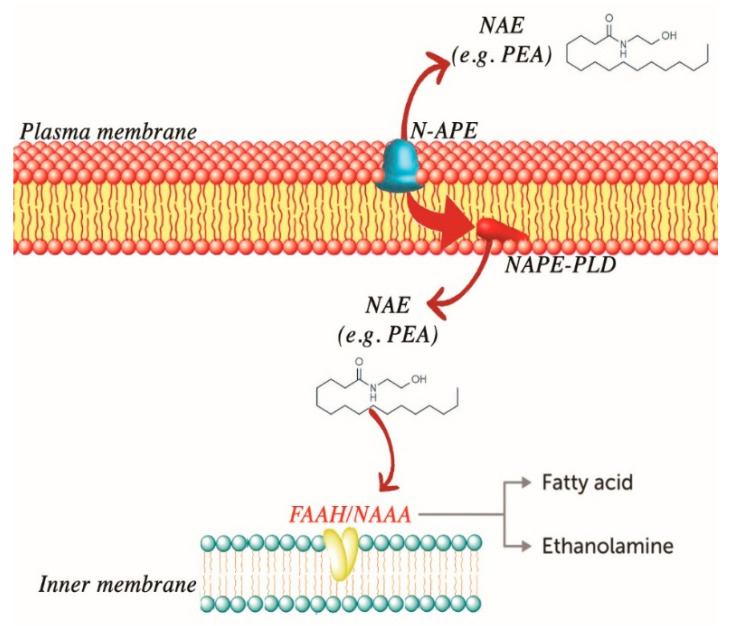
Biosynthesis and degradation of palmitoyl ethanolamide (PEA).

The biosynthesis of PEA occurs through a common enzyme for the other NAEs, the selective phospholipase N-acyl-phosphatidyl-ethanolamine D (NAPE-PLD), starting from the hydrolysis of the precursor N-palmitoyl-phosphatidyl-ethanolamine (NAPE) [119]. PEA degradation occurs through the action of two separate enzymes: the first specific for PEA, the amidase of N-acylethanolamine-hydrolysing acid (NAAA), while the second, the FAAH enzyme, deputy to the hydrolysis of PEA and the other NAEs [111,116,117,120]. See Figure 1.

Three mechanisms have been proposed so far to explain the anti-inflammatory and analgesic effects of PEA. The first mechanism, which does not exclude the other two, suggests that PEA acts via an ‘ALIA’ mechanism; hence, the name of this family of molecules, according to which PEA acts by down-regulating mast-cell degranulation [97,110]. Furthermore, many studies demonstrated that PEA acts via the direct activation of two different receptors: the nuclear peroxisome proliferator-activated receptor-a (PPAR-α) [121] and the orphan receptor G-protein coupling (GPR55) [122]. In particular, PPAR-α is one of a group of nuclear receptor proteins that function as transcription factors that regulate the expression of genes and it is associated with pro-inflammatory effects [55]. Moreover, in PPAR-α KO mice or mice with blocking PPAR-α antagonists, the anti-inflammatory, anti-nociceptive/anti-neuropathic, and neuroprotective effect of PEA was not detected [123]. Finally, an “entourage effect” was also proposed to explain PEA’s pharmacological activities regarding improving the anti-inflammatory and anti-nociceptive function of other endogenous substances through potentiating their receptor binding or inhibiting metabolic degradation. In fact, PEA acts by indirectly activating CB2 and CB1 receptors cannabinoid or transient receptor potential vanilloid receptor type 1 (TRPV1) channels [124], probably by increasing the levels of AEA and 2-AG, for example by inhibiting the expression of FAAH, the enzyme that is responsible for their degradation [125]. Additionally, the anti-inflammatory effect can be obtained by upregulating endogenous PEA levels and targeting its major catabolic enzyme, NAAA, through the modulation of its degradation [126]. In the native state, PEA presents lipid structure and the large size of heterogeneous particles, so it may be expected to have limitations in terms of solubility and bioavailability. Micronization and ultramicronization represents a potential solution for bypassing this problem. The micronization method is applied to reduce particle size (< 10 μm) and increase the bioavailability and efficacy of low water-soluble molecules, so increasing the dissolution rate [101,127]. Impellizzeri et al. [106] tested these PEA formulations in carrageenan-induced inflammation in rat paw—a classic model of oedema formation and hyperalgesia widely used in the development of anti-inflammatory drugs—using the air-jet milling technique to produce micronized (m-PEA) and ultramicronized PEA (um-PEA). These formulations, in comparison to a non-micronized PEA preparation, had superior pharmacological action against carrageenan-induced inflammatory pain. Protective and antiinflammatory effects of m-PEA and um-PEA were also observed in other inflammatory disorders [128,129,130,131,132].

2-pentadecyl-2-oxazoline (PEA-OXA) is a new form of PEA that, besides having the classic anti-inflammatory, analgesic, and neuroprotective properties of the latter, also has the ability to modulate the catalytic activity of NAAA, which is responsible for PEA degradation. It has been observed that PEA-OXA has a greater efficacy in reducing inflammation and hyperalgesia when compared to PEA [133], probably due to pharmacological modulation of NAAA. Moreover, PEA-OXA has been shown to improve the behavioural assessment that is associated with biochemical alterations and neuroinflammation [134,135,136].

Additionally, it has been widely demonstrated that the anti-inflammatory and protective action of PEA combined with antioxidant molecules by co-micronization process could potentiate its pharmacological effects. Among the natural compound, there are numerous flavonoids, such as Baicalein, Luteolin, Polydatin, and Silymarin, which have different pharmacological and therapeutic actions [137,138,139,140,141,142,143]. Among these, quercetin co-ultramicronized with PEA has attracted particular attention in pain management. In fact, Britti et al. showed that the association between quercetin and PEA in co-ultramicronized form exerts beneficial effects in both inflammatory and mixed persistent OA pain in rats [144].

### 4.2. OEA and SEA

As mentioned above, OEA and SEA, together with PEA, belong to the family of N-acyl ethanolamines (NAEs), present in both plant and animal tissues. Like PEA, OEA and SEA are produced on demand through NAPE-PLD and they are rapidly catalyzed by enzymatic hydrolysis, suggesting a function in cellular signalling [145]. Although structurally and functionally related to endocannabinoids, these compounds do not bind to cannabinoid receptors [146]. OEA presents high affinity to the nuclear receptor PPAR-a [147]; whilst, SEA has been proposed to activate PPAR-γ [148]. Several evidences suggest that these bioactive lipids are involved in many physiological processes that are directly linked with the maintenance of gut-barrier function, the regulation of inflammation and pain, and energy metabolism. OEA and SEA principally regulate food intake and metabolic pathways [149,150]; however, emerging evidence proposes that NAEs appear to play major roles in the modulation of pain sensitivity and inflammatory processes [151,152]. In particular, it has been shown that OEA may participate in the peripheral nociceptive pathway, perhaps modulating or modifying both the altered C-fibre activation and/or the inflammatory process in the peripheral tissues. [152,153]. However, there is no complete literature on the role of the OEA and SEA in controlling pain, but they are recently raising great interest in the treatment of several chronic inflammatory disorders, including neuropathic pain [104,152].

### 4.3. Adelmidrol

Together with PEA, other compounds that belong to the ALIAmides family have been described, including Adelmidrol (N,N′-bis (2-hydroxyethyl) nonanediamide). It is a di-ethanolamide derivative of azelaic acid, which is found in nature in some whole grains and trace quantities in the human body [154]. Chemically, Adelmidrol possesses both hydrophilic and hydrophobic properties that favour its solubility both in aqueous and organic media [154]. These properties make it especially suitable for topical application; in fact, recently, an emulsion of adelmidrol (2%) has shown some benefit in a pilot study on mild atopic dermatitis [155]. As PEA analogue, the pharmacological properties of Adelmidrol can be related to its ability to down-modulate mast cells activation and mast cell mediators release [156]. The role of MCs in chronic inflammation-induced hyperalgesia has been well documented. For example, De Filippis et al. [154] have shown that mast cell mediators, released at early stage of the inflammatory process, play a pivotal role in a classical model of chronic inflammation, i.e., the λ-carrageenin-induced granuloma formation [157]. On contrary, previous studies have clearly established that adelmidrol presents some important differences when compared to PEA. In particular, it has been demonstrated that adelmidrol unlike than PEA, exerts its anti-inflammatory properties by action only to the PPARγ receptor, but not PPARα or CB2 related pathways [158,159]. Furthermore, also Adelmidrol exerts a physical effect, known as “entourage effect”, which causes a substantial endogenous increase of local levels of PEA, enabling the preservation of normal reactivity of the mast cells [160]. For this reason, in the last years, Adelmidrol has been considered as a successfully treatment for inflammatory disease showing great efficacy in the treatment of pain and inflammation comparable to PEA in studies in vivo and in vitro [159,161,162,163,164,165]. In that regard, Impellizzeri et al. [105] showed that adelmidrol treatment significantly reduced thermal hyperalgesia and mechanical allodynia during both acute (CAR model) and chronic inflammation (CIA model). They also demonstrated that adelmidrol was able to ameliorate peripheral sensitization (altered heat sensitivity) and central sensitization (mechanical hypersensitivity). Additionally, some works demonstrated the beneficial effects of adelmidrol in combination with sodium hyaluronate [160,166], in particular in a model of arthritis pain that is associated with osteoarthritis (OA). Because pain is the predominant symptom of OA, it was assessed that adelmidrol was able to reduce pain and tactile allodynia, improving joint mobility and locomotor functionality [164]. Thus, local analgesic and anti-inflammatory effects observed with adelmidrol treatment could be useful in the treatment of inflammatory diseases that are associated with pain.

### 4.4. Glupamid

N-Palmitoyl-d-glucosamine (PGA, also referred to as Glupamid^®^) is one of the less studied among ALIAmides, and only few reports on this compound have been published so far [167,168]. Chemically, PGA is the amide of palmitic acid and glucosamine [167]. Similarly to fatty acid amides, it is hydrolysed by fatty acid amide hydrolases [169], resulting in the intracellular release of glucosamine. Thus, PGA might exert a dual effect, i.e., the characteristic chondroprotective effects of glucosamine [170,171], but also the anti-inflammatory/anti-nociceptive activities of aliamides. In particular, PGA down-modulates MC degranulation—the typical mechanism of ALIAmides [99]. It is interesting to note that allodynia, which is a peculiar feature of neuropathic pain, is possibly related to OA [172,173]. Accordingly, the effect of PGA in a reliable animal model of osteoarthritis pain (i.e., the intraarticular injection of monosodium iodoacetate, MIA) [174] has been investigated. The administration of PGA resulted in a significant relief of mechanical allodynia accompanied by a reduction in inflammation and MC activation [113]. In addition, it has recently been demonstrated that micronized PGA (m-PGA; particle size from 0.6 to 10 μm) resulted in a superior activity to PGA on MIA-induced mechanical allodynia, locomotor disability, and on chondrodegeneration and inflammation [167]. The superior effect of the tested micronized formulation is in agreement with what has been said above, about PEA. The reduction of particle size highly and significantly increased both bioavailability and pain-relieving effect [175,176]. Finally, a novel compound developed by co-micronizing PGA and curcumin was found to regulate pain sensitivity, reducing allodynia, joint pain, and improving locomotor function in MIA-injected animals [113]. Clinical studies in human and veterinary patients are warranted to further evaluate therapeutic potential for PGA and m-PGA in in management of pain.

## 5. ALIAmides in Peripheral Neuropathic Pain

Now, we report the in vitro and in vivo findings, along with clinical results, supporting the possible role of ALIAmides, in particular PEA the most prominent among ALIAmides, as a therapeutic agent in peripheral pain.

Damage to peripheral nerve is often due to compression and cutting and through a variety of trauma, or ischemic and metabolic disorders. This produces a condition of neuropathic pain, characterized by an increase in painful sensitivity, such as hyperalgesia and allodynia. Moreover, the compression of the peripheral nerves is often also associated with the loss of motor function, mainly due to an insufficient regeneration of the nerve. Additionally, nervous injury initiates a cascade of events, including the degeneration of the distal part of the nerve, endoneural edema and increased of cells infiltration, which form part of a complex mechanism, called as Wallerian degeneration [177].

Costa and colleagues [178] have suggested that PEA was able to reduce mechanical allodynia and thermal hyperalgesia following sciatic nerve constriction in mice, through an action upon receptors located on the nociceptive pathway. Additionally, the same research team showed that PEA induces relief of neuropathic pain probably through a more direct action on an exclusive target, namely the mast cells, via the ALIA mechanism. In details, their studies have demonstrated that, after nerve injury, there was no further increase in mast cell number, but rather a marked activation of these cells, with a high ratio of degranulated to non-active cells [179]. The evaluation of mast cells in the sciatic nerve of CCI (chronic constriction injury) mice treated with PEA clearly indicates that this compound is able to significantly delay the recruitment of MC in the early phase of neuropathic pain caused by nerve injury and inhibit their degranulation during the subsequent phase [179]. Because numerous mediators that are released by MC contribute to the degeneration of myelinated fibers [180], it has been showed that CCI is often accompanied by a local inflammatory reaction, which includes endoneural edema, disorder of nervous architecture, and infiltration of immune cells. On the other hand, the treatment with PEA attenuates the degree of peripheral inflammation, reducing edema and macrophage infiltration allowing for hypothesizing a synergism between the anti-inflammatory and the neuroprotective mechanisms of PEA [179]. The efficacy of PEA to modulating neuropathic pain is consistent with the previous study conducted by Petrosino et al. [181], which observed a decrease in endogenous PEA rates in the spinal cord and in areas of the brain directly or indirectly involved in nociception in CCI rats. The efficacy of PEA in attenuating neuropathic pain is consistent with the study by Guida et al. [182]. In their study, animals at 30 days after spared nerve injury (SNI) showed mechanical and thermal hypersensitivity, together with a late development of anxio-depressive syndrome. Daily treatment with the acylethanolamide PEA (or OEA) reduced most of pain symptoms in a model of debilitating long lasting pain (the SNI model), which is comparable to the advanced stage of neuropathy in humans [183]. Similarly, Boccella et al. [184] found that also the ultra-micronized form of PEA was able to ameliorate significantly pain, mechanical allodynia and thermal hyperalgesia in an experimental model of spared nerve injury in mice, induced by ligature with 5.0 silk thread around sciatic nerve. Furthermore, in a recent study, Gugliandolo and colleagues [185] have investigated the effects of PEA-OXA on pain inhibition and pathological processes after crush to the sciatic nerve through use of an ultra-fine, smooth, straight hemostat (tip width 0.6 mm) for 30 s. In the latter study, the daily administration of PEA-OXA showed a significant reduction of endoneural edema and the number of mast cells, as well as a reduction of degeneration of the nerve structure. Accordingly, overall, the treatment with PEA-OXA had a marked analgesic effect, inhibiting the mechanical allodynia and thermal hyperalgesia. Moreover, in a recent study, the association of um-PEA, together with paracetamol, a potent analgesic compound, showed a synergistic effect on pain inhibition and pathological processes after crushing of the sciatic nerve [186]. These findings support the hypothesis that PEA-induced relief of neuropathic pain might be attributed, at least in part, to the ability of this compound to modulate mast cell recruitment in the sciatic nerve and activation of microglia in the spinal cord. In this way, combining this dual activity on both nociceptive pathway neurons and the modulation of non-neuronal cells, PEA, and its formulations could offer more benefits than traditional anti-nociceptive drugs, so representing an innovative molecule for the treatment of pain, like neuropathic one.

In addition, recently, some studies have investigated the action of PEA in diabetic peripheral neuropathy (DPN), a type of peripheral neuropathic pain. DPN is common, long-term complication of type 1 or type 2 diabetes mellitus, encompassing a broad spectrum of clinical and pathophysiological frameworks that affecting PNS [187]. In this context, the etiology is multifactorial and pathogenic factors include paraesthesias, sensory loss, motor deficits, and severe neuropathic pain (burning, lancinating, tingling, or shooting) that seriously compromises the quality of life of patients [188,189,190]. A typical experimental model of diabetic neuropathy is represented by streptozotocin (STZ)-injection induced autoimmune diabetes in rodents. Single or repeated injection of STZ induces diabetes together with hyperglycemia and renal biochemical alterations. This produces significant damage in sciatic nerve tissue, which is followed by hyperalgesia and allodynia [191,192]. Collectively, the findings in two independent experimental studies [193,194] propose that PEA and its micronized form, PEA-m, was able to decrease damage in sciatic nerve tissue after DPN-induced. Moreover, PEA and PEA-m reduce fiber degeneration, endoneurial edema, and MC activation. Consequently, it led to a reduction of thermal mechanical hyperalgesia and pain sensitivity, improving motor activity.

The efficacy of PEA, after acute or repeated treatment, was also highlighted in a preclinical model of oxaliplatin-induced neuropathy [195]. Painful chemotherapy-induced neuropathy can remain from months to years after completion of chemotherapy, presenting significant challenges for cancer survivors due to negative impact on quality of life [196,197]. Neurotoxicity may result in chemotherapy dose reductions or early discontinuation [198]. In details, PEA substantially decreased oxaliplatin-dependent pain, when measured as an increase upon suprathreshold stimulation (measurement related to hyperalgesia) or as a decrease in pain threshold (measurement related to allodynia) [195]. Donvito and colleagues [199] observed similar results in an experimental model of paclitaxel-induced neuropathy in mice. In both cases, the mechanisms by which the administration of PEA produced antiallodynic, analgesic, and neuroprotective effects may be linked with a direct action on MC, via autacoid local injury antagonist mechanism [97], combining the dual activity of neurons in nociceptive pathways and non-neuronal cells, such as MC in the periphery and microglia in the spinal cord.

The temporomandibular joint (TMJ) syndrome, also known as temporomandibular disorder (TMD), is a common type of disorder in the orofacial region leading to severe pain and a limitation of the jaw range of motion. The sensory nerve supply to the TMJ is by the auriculotemporal and masseteric branches of the mandibular nerve (V3), which is a branch of the trigeminal nerve [200]. Pain may range from sudden, severe, and stabbing to a more constant, aching, burning sensation [201,202]. Two separate experimental researches demonstrated the antinflammatory and analgesic effects of PEA on trigeminal hypersensitivity and m-PEA on temporomandibular joint (TMJ) pain. The ability of PEA to modulate the mechanical sensitization of peripheral trigeminal nerve endings has been observed when diethylenetriamine was inoculated into the trigeminal ganglion in vitro and in vivo [203]. Additionally, Bartolucci et al. [204] showed that the micronized form of PEA significantly reduced inflammation and pain, as evidenced by the reduction in edema and mechanical allodynia after the injection of complete Freund’s adjuvant (CFA) emulsion into the left TMJ capsule.

Complex regional pain syndrome (CRPS) is a rare neuropathic pain disorder that is associated with severe pain [205]. CPRS typically occurs after a regional injury and presents nociceptive, vascular, and bone distal limb changes that surpass the predicted clinical course of the initiating injury in both severity and duration, often resulting in severe motor dysfunction and disability. CRPS symptoms typically gradually improve over the first year after injury, but chronic CRPS is a severe concern, resulting to edema, periarticular bone loss, pain, and allodynia in the injured limb [206]. The most widely used CRPS model is a rodent tibia fracture model (TFM) [206] that duplicates the most common etiology of CRPS, distal limb fractures [207]. After distal tibia fracture and casting the hindlimb for four weeks, most rodents develop chronic ipsilateral hindpaw warmth, edema, mechanical allodynia, hindlimb unweighting, and periarticular osteoporosis. These symptoms occur primarily in the injured limb, but some changes are also observed in the contralateral limb [208]. Fusco and colleagues [209], in their study, assessed the effects of a formulation of micronized and ultramicronized PEA (PEA-MPS), given orally in a mouse model of CRPS-I. The findings described by them demonstrated that 28 days after tibia fracture induction, PEA-MPS was able to attenuate the inflammatory response and MC activation, allodynia, and hyperalgesia induced by tibia fracture.

Taken together, PEA and its congeners could represent an innovative therapeutic strategy in the management of mixed neuropathies.

### Clinical Studies

In addition, has been repeatedly shown that PEA, particularly in the micronized and ultra-micronized form, relieves in neuropathic pain also in human patients. Treatment with different PEA formulations resulted in a major reduction of pain symptoms, as demonstrated by a number of clinical trials.

In details, nerve pressure induces pain and inflammation of nerves and nerve roots, neuritis, and radiculitis. Subsequently, they progress into a more chronic pathological state due to the induction of a number of cascades of chemical inflammatory reactions [210], with recruitment of inflammatory cells, such as activated mast cells. The most common compression neuropathy is carpal tunnel syndrome (CTS) and it is due to the compression of the median nerve in the passage through the carpal tunnel in the wrist [211]. The compression of the median nerve causes pain, numbness, and tingling in the thumb, index finger, middle finger, and the thumb side of the ring finger. Symptoms typically start gradually and may extend up the arm [212]. As regards radicular pain, described as radiculopathy, is frequently secondary to compression or inflammation of spinal nerve roots [213]. Radiculopathy is much worse than low back pain, and the specific areas of the leg and/or foot that are affected depend on which nerve in the low back is affected [214]. Pain can radiate down the back along a leg or foot, and this is described as sciatica, which can usually be reproduced with certain activities and positions, such as sitting or walking, and it is often caused by the compression of the lower spinal nerve roots (L5 and S1) [215]. Overall, several findings would suggest that PEA and PEA-um administration might be effective in the management of pain sciatic associated with lumbosciatalgia [216,217] or lumbar radiculopathy [218], and of pain associated to carpal syndrome [211,219,220]. Moreover, according to the literature, patients complain of pain and swelling as the main factors negatively influencing their quality of life [221] during postoperative course, as well as in patients suffering from neuropathic pain that is associated with pathologies of various etiologies [222], such as post-herpetic neuralgia. PEA and its formulations have been successfully used in patients who were unable to effectively control chronic pain with standard therapies [223], postoperative pain [224], polyneuropathic [225], and diabetic patients [226,227]. Finally, recent observational clinical studies reported the beneficial use of um-PEA as an add-on therapy in patients suffering from low back pain [228] as well as in patients suffering from fibromyalgia syndrome [229]. In particular, fibromyalgia syndrome is a chronic multifaceted disease that is characterized by widespread pain, stiffness of muscle, tendons, and joints, fatigue, and cognitive disorders [230]. A recent retrospective analysis shown the results of um-PEA treatment in pain management during fibromyalgia syndrome, which suggest it as a new and well-tolerated therapy, mostly suitable for patients who need long-term treatments [229].

It is good to underline that PEA induced pain relief is progressive, age- and gender independent, and is not related to etio-pathogenesis of chronic pain [231]. This supports the view that PEA controls mechanisms common to different conditions where chronic pain and neuropathic pain is associated, e.g., neuroinflammation. Importantly, PEA lacks acute and chronic toxicity, and it is well-tolerated and no interaction with other ongoing therapy was reported [115].

## 6. Conclusions

**Figure 2:**
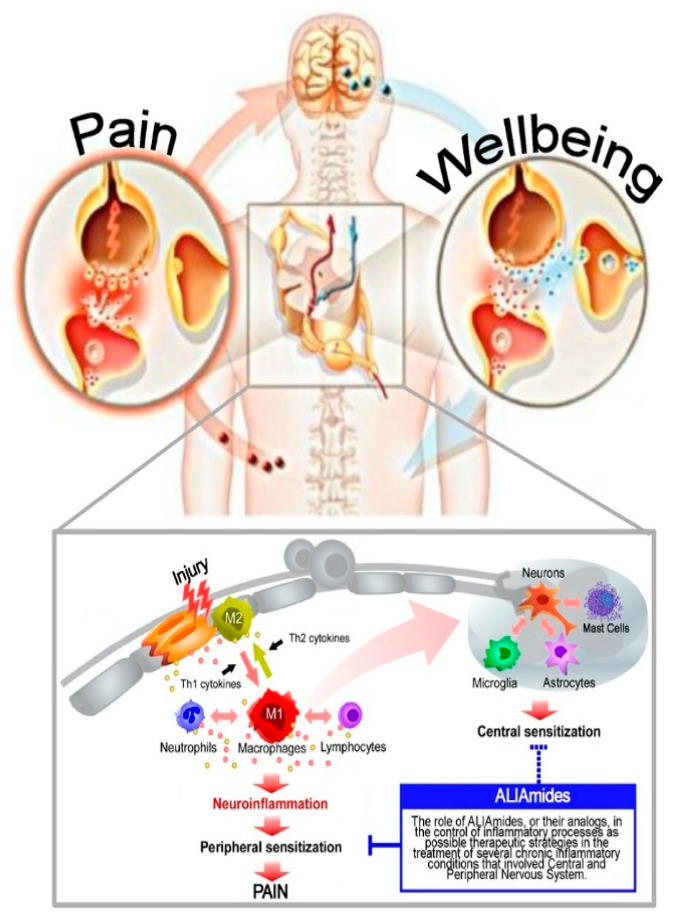
Effects of ALIAmides in attenuating pain.

Peripheral neuropathic pain is a very common condition and it remains one of the most difficult diseases to treat. This is probably due to the multiple signalling mechanisms underlying pain transmission (Figure 2). As mentioned previously, a greater knowledge of the role of neuroinflammation in neuropathic pain could open new perspectives for therapies aimed at modulating the activation of neuronal and non-neuronal cells that normally control neuronal sensitization. Currently, drug therapies in treating neuropathic pain involve the use of opioids, tricyclic antidepressants, and anti-convulsants, which exhibit a wide spectrum of adverse side effects. Hence, research is focused on identifying alternative therapies with less side effects. The present review sheds light on the effects of ALIAmides in attenuating pain, in particular peripheral neuropathic pain. The capacity of ALIAmides to exert antiallodynic and anti-hyperalgesic effects by down-modulation both microglial and mast cell activity has led to the hypothesis that these compounds could represent an innovative therapeutic strategy for the treatment of all conditions that are characterized by the presence of neuroinflammatory processes and chronic painful states.

## Figures and Tables

**Table 1:** The more common neuropathic pain.

Central Neuropathic Pain	Peripheral Neuropathic Pain
Spinal cord injury	Diabetic painful neuropathy (DPN)
Central post-stroke pain	Alcoholic neuropathy
Parkinson disease pain	Cancer pain
Multiple sclerosis-related pain	Chemotherapy-induced peripheral neuropathy (CIPN)
Transverse myelitis	Charcot-Marie-Tooth disease
Neuromyelitis optica	Trigeminal neuralgia
Syringomyelia	Acute e chronic inflammatory demyelinating polyneuropathy
	Human immunodeficiency virus-associated neuropathy
	Post-traumatic neuropathy
	Phantom limb pain Compressive mononeuropathies and many more.

**Table 2:** Cellular and intracellular elements involved.

Ion Channels	*Na, Ca, K*
Ionotropic and metabotropic receptors	Glutamatergic, *GABA (γ-aminobutyric acid) ergic, serotoninergic, adrenergic*
Inflammatory cytokines	*IL-1β, IL-6, TNF-α*
Nerve growth factors	*NGF*
Intracellular regulators	*protein kinase C*
Transcriptional factors	*nuclear factor-κB*

**Table 3:** Allodynia and Hyperalgesia.

Allodynia	Hyperalgesia
refers to pain produced by a normally non-painful stimulus, and it may result from decreased stimulation thresholds	refers to exaggerated pain perception as a result of damaged peripheral pain fibers
Classified: -mechanical (pain in response to light touch)—thermal (hot or cold: pain from normally mild skin temperatures in the affected area) -movement pain triggered by normal movement of joints or muscles	Classified: -primary hyperalgesia: occurs directly in injured tissue as a result of sensitization of peripheral nociceptors (for example, tenderness after a cut), -secondary hyperalgesia: occurs in adjacent undamaged tissue owing to sensitization within the CNS
An example is a patient with diabetic neuropathy whose feet are sensitive to putting on socks.	A clinical example of hyperalgesia might be an amputee who is unable to use a prosthesis because of tenderness overlying the stump.

**Table 4:** Major protagonists involving during neuroinflammatory events.

Protagonists	Function	References
Astrocytes	Involved in brain homeostasis, provide metabolites and growth factors to neurons, support synapse formation and plasticity, participate in BBB maintenance and permeability.	[61,62,63]
Oligondencytes	Involved in the formation of myelin, propagation of action potentials along axons, production of neurotrophic factors that support to neurons	[55,64]
Inflammasomes	Involved in induction of the pyroptosis process, responsible for the secretion of the inflammatory cytokines	[65,66]
Cytokines and chemokines	Involved in tissue repair and homeostasis restoration, responsible to cell migration	[67,68]

**Table 5:** Pharmacotherapies in use in neuropathic pain.

Drug Class		Kind of Neuropathic Pain	Effects	**Side Effects**	**References**
*First-Line Drugs*					
Antidepressants	-Tricyclic antidepressants (TCAs): *amitriptyline, nortriptyline, desipramine,* *imipramine* -Serotonin-norepinephrine reuptake inhibitors (SNRIs): *Duloxetina* *Venlafaxina*	Painful diabetic neuropathy Post-herpetic neuralgia Post-stroke pain Painful polyneuropathy Low back pain	Inhibition the reuptake of serotonin and noradrenaline into the spinal synapses between nociceptors (or first-order neurons) and the spinothalamic neurons (or second-order neurons)	Sedation Heart problems Constipation Drowsiness Light-headedness Weight gain Dry mouth Nausea (SNRIs less side effects than TCAs)	[76,77,78]
Anticonvulsants	-Phenytoin -Gabapentin -Carbamazepine -Oxcarbazepine -Valproic acid	Lancinating pain and allodynia Painful diabetic neuropathy Trigeminal neuralgia Post-herpetic neuralgia Painful polyneuropathy Low back pain	Reduction of neuronal excitability and local neuronal discharges, acting through sodium channel blockade or modulation of calcium channels	Dizziness Somnolence Skin reactions such as Stevens–Johnson syndrome Leukopenia Hyponatremia	[79,80,81,82]
*Second- and Third-Line Drugs*					
Topical agents	-Lidocaine -Capsaicin -Clonidine -EMLA (eutectic mixture of local anesthetics)	Allodynia Post-herpetic neuralgia Chemotherapy-induced peripheral neuropathy Post-surgical and post-traumatic neuropathic pain	Block of voltage-gated sodium channels expressed by nerve fibers, responsible for the propagation of action potentials.	Local irritation Possible hypersensitivity	[75,83,84,85,86]
Opioids	-Morphine -Oxycodone, -Hydromorphone -Tramadol	Diabetic peripheral neuropathy Post-herpetic neuropathy Polyneuropathy Phantom limb pain	Opioid receptors are coupled to calcium and potassium channels, block synaptic transmission, restricting the number of nociceptive stimuli	Drowsiness Nausea Dependence Overdoses	[76,87,88,89]
Corticosteroids	-Prednisone -Desametasone	Allodynia Spinal cord compression Post-herpetic neuralgia	Inhibition of prostaglandin synthesis, reduction inflammation, vascular permeability and tissue edema	Gastrointestinal disease Psychiatric disorders Electrolyte imbalances Bone demineralization	[90,91]
Alternative non-pharmacologic therapies	-Acupuncture -Magnetic insoles -Repetitive transcranial magnetic stimulation (rTMS)	Chemotherapy-induced peripheral neuropathy Trigeminal neuralgia Post-stroke pain Post-herpetic pain	Local inhibition of nociceptive fibres; stimulates blood flow to restore nerve damage.	Bruising Infection	[92,93]

## Abbreviations

ALIAmides	Autacoid local injury antagonist amides
BBB	Blood–brain barrier
BSCB	Blood-spinal cord barrier
CCI	Chronic constriction injury
CIPN	Chemotherapy-induced peripheral neuropathy
CNS	Central nervous system
DPN	Diabetic painful neuropathy (DPN)
GPR	Receptor G-protein coupling
IASP	International Association for the Study of Pain
MAPK	Mitogen-activated protein kinase
MC	Mast cell
m-PEA	micronized-PEA
NAAA	N-acylethanolamine-hydrolyzing acid amidase
NAEs	N-acyl ethanolamines
OEA	Oleoyl ethanolamide
PEA	Palmitoyl ethanolamide
PEA-OXA	2-pentadecyl-2-oxazoline
PGA	N-Palmitoyl-d-glucosamine
PNI	Peripheral nerve injury
PNS	Peripheral nervous system
PPAR-α	Peroxisome proliferator-activated receptor-α
ROS	Reactive oxygen species
SEA	Stearoyl ethanolamide
SNI	Spared nerve injury
SNRIs	Serotonin-norepinephrine reuptake inhibitors
STZ	Streptozotocin
TRPV1	Transient receptor potential vanilloid receptor type 1
TCAs	Tricyclic antidepressants
um-PEA	Ultramicronized-PEA
